# Temporal trends in Black‐White disparities in cancer surgery and cancer‐specific survival in the United States between 2007 and 2015

**DOI:** 10.1002/cam4.5141

**Published:** 2022-08-15

**Authors:** Peijie Zhong, Bo Yang, Feng Pan, Fang Hu

**Affiliations:** ^1^ Clinical Medical College Southwest Medical University Luzhou China; ^2^ Department of Gastroenterology and Hepatology Huaihe Hospital of Henan University Kaifeng China; ^3^ Department of Interventional Medicine The Affiliated hospital of Southwest Medical University Luzhou China; ^4^ College of nursing Southwest Medical University Luzhou China

**Keywords:** disparity research, health inequity, racial disparity, SEER database, surgical oncology

## Abstract

**Background:**

The American Society of Clinical Oncology (ASCO) has strived to address racial/ethnic disparities in cancer care since 2009. Surgery plays a pivotal role in cancer care; however, it is unclear whether and how racial/ethnic disparities in cancer surgery have changed over time.

**Methods:**

This cohort study included 1,113,256 White and Black cancer patients across 9 years (2007–2015) using patient data extracted from the Surveillance, Epidemiology, and End Results (SEER)‐18 registries. Patient data were included from 2007 to adjust insurance status and by 2015 to obtain at least a 3‐year survival follow‐up (until 2018). The primary outcome was a surgical intervention. The secondary outcomes were the use of (neo)adjuvant chemotherapy and cancer‐specific survival (CSS). Adjusted associations of the race (Black/White) with the outcomes were measured in each cancer type and year.

**Results:**

The gap between surgery rates for Black and White patients narrowed overall, from an adjusted odds ratio (aOR) of 0.621 (0.592–0.652) in 2007 to 0.734 (0.702–0.768) in 2015. However, the racial gap persisted in the surgery rates for lung, breast, prostate, esophageal, and ovarian cancers. In surgically treated patients with lymph node metastasis, Black patients with colorectal cancer (CRC) were less likely to receive (neo)adjuvant chemotherapy than White patients. Black patients undergoing surgery were more likely to have a worse CSS rate than White patients undergoing surgery. In breast cancer patients, the overall trend was narrow, but continuously present, with an adjusted hazard ratio (aHR) of 1.224 (1.278–1.173) in 2007 and 1.042 (1.132–0.96) in 2015.

**Conclusions:**

Overall, progress has been made toward narrowing the Black‐White gap in cancer surgical opportunity and survival. Future efforts should be directed toward those specific cancers for which the Black‐White gap continues. Additionally, it is worth addressing the Black‐White gap regarding the use of (neo)adjuvant chemotherapy for CRC treatment.

## Graphical abstract

In this study, using a large SEER based data, we examined whether and how racial/ethnic disparities in cancer surgery and outcome have altered after 2009 for overall and 11 deadliest cancers in the US. We found overall significant progress has been made in narrowing the black‐white gaps in cancer surgical opportunity and survival over time. " cd_value_code="text

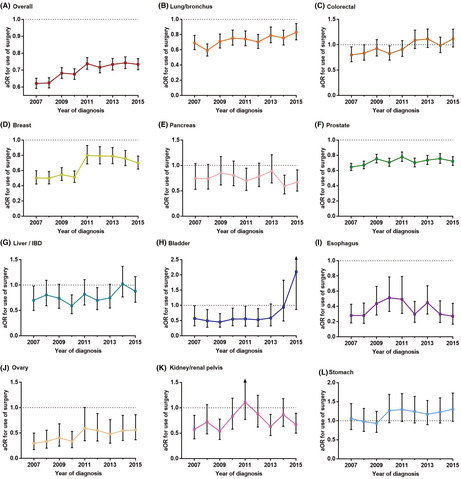

## 1 INTRODUCTION

Racial/ethnic disparity is one of the most pronounced cancer‐specific disparities in the United States (US).^1^, ^2^ For example, the cancer stage at diagnosis, treatment, and survival varies by race/ethnicity.^3^ The racial/ethnic disparity is rooted in historical and modern times and is associated with different causes, such as socio‐demographic factors and structural racism.^4^ When compared to the white majority, US minorities, including Black, Hispanic, Asian/Pacific Islander, and American Indian/Alaskan Native, are often at a disadvantage regarding cancer care.^5^ Among them, the Black‐White disparity remains the most acute. For instance, recent studies have suggested that Black patients with colorectal cancer (CRC) or breast cancer have an increased risk of being diagnosed at an advanced stage of illness and experience a decreased survival rate compared to White patients. This disparity is considered a consequence of racial residential segregation.^6^, ^7^ Prior civilian research has found that Black individuals with early‐stage lung cancer are less likely to receive surgery. Even when Black patients do undergo surgery, they experience poorer outcomes, including a considerably shorter overall survival rate, than White patients.^8^, ^9^ Furthermore, Shavers et al.^10^ discovered that ethnic minorities, especially Black patients, have a 33% greater chance of dying from cancer than White patients. Factors that may play a role in racial differences are multilayered and multidimensional, including policy‐related obstacles (e.g., inadequate insurance coverage), quality‐of‐care access (e.g., poorer surgical performance), and literacy of health care providers (e.g., discrimination and bias among providers).^11^ Given that cancer is a broad public health problem, it is critical to examine the opportunities for vulnerable patient groups to access surgeries.

Surgery is a pivotal cancer treatment and largely determines a patient's long‐term prognosis. Racial/ethnic disparity in the surgical treatment of cancer is a persisting problem and was recently highlighted by the American Board of Surgery (ABS).^12^, ^13^ In 2009, the American Society of Clinical Oncology (ASCO) listed racial/ethnic disparity as a critical issue in achieving broad cancer health equity.^4^, ^14^ It is essential to fully examine the cancers which are associated with unequal surgical opportunity and survival rates due to ethnicity/race. Therefore, in this population‐based, cohort study, we enrolled 11 common cancers across 9 years and combined them into a pan‐cancer atlas. We aimed to generally and specifically examine the US Black‐White disparities regarding cancer surgery rates and cancer‐specific survival (CSS) time.

## 2 METHODS

### 2.1 Database and study population

**FIGURE 1:**
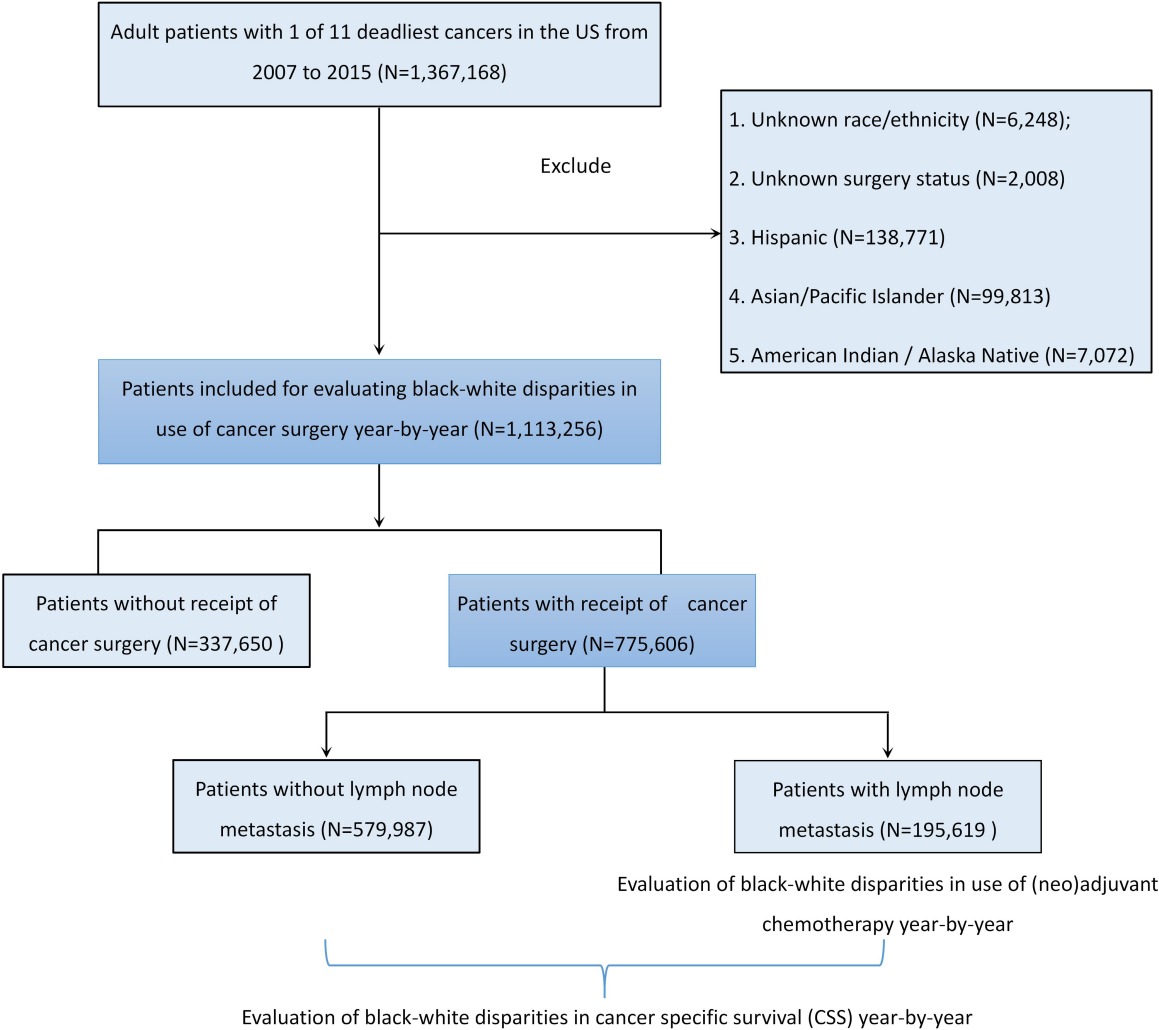
Flowchart of the study population selection.

This retrospective, cohort study used custom data (with additional treatment fields) retrieved from the Surveillance, Epidemiology, and End Results (SEER)‐18 registry database, based on the November 2018 submission. The SEER program collects data on different cancers from population‐based cancer registries, covering approximately 28% of the US population. The data use agreement was signed, and data were extracted using the SEER*Stat software v. 8.3.8 (https://seer.cancer.gov/seerstat/software/). According to SEER statistics (https://seer.cancer.gov/statistics/), the 11 deadliest solid cancers were selected: lung/bronchus, colorectal, breast, pancreatic, prostate, liver/intrahepatic bile duct (IBD), bladder, esophageal, ovarian, kidney /renal pelvic, and stomach cancers. The inclusion criteria were as follows: (1) patients diagnosed via positive histology with 1 of the 11 cancers selected; (2) patients diagnosed between 1 January 2007 and 31 December 2015 (patients were included in 2007 to adjust insurance status and by 2015 to obtain at least 3‐year survival follow‐up until 2018); (3) the cancer was primary cancer only or the original of 2 or more primaries; (4) patients aged ≥18 years; (5) patients with known information on age, gender, marital status, insurance type, the American Joint Committee on Cancer (AJCC) tumor—node—metastasis (T, N, and M) stages and cause of death (COD) with follow‐up survival in months. The exclusion criteria were as follows: (1) patients with unknown race/ethnicity; (2) patients with unknown surgery status; and (3) Hispanic, Asian/Pacific Islander, and American Indian/Alaska Native patients. Finally, 1,113,256 White and Black cancer patients in the US spanning over 9 years were included in this study. The recruitment flowchart is shown in Figure 1.

### 2.2 Exposure, covariates, and outcomes

The exposure was race, divided into White and Black.^15^ The primary outcome was a surgical intervention. Association of the race with surgery use was measured in each cancer and year, after adjusting for age, gender, marital status, insurance status, and AJCC T, N, and M stages. In surgically treated patients with positive lymph node (LN) metastasis (AJCC N stage ≥1), associations of race with the use of (neo)adjuvant radiotherapy and chemotherapy were independently measured in each cancer and year, after the same adjustments mentioned above. In patients with surgery, the association of race with CSS was independently measured in each cancer and year after adjusting for age, gender, marital status, insurance status, AJCC T, N, and M stages, and use of (neo)adjuvant radiotherapy and chemotherapy.

### 2.3 Statistical analysis

Age was expressed as means with standard deviations (SD) and examined by the Student's t‐test. The categorical variables were expressed as numbers with percentages and examined by the chi‐square (χ^2^) test. Race associations with the use of surgery, (neo)adjuvant radiotherapy, and chemotherapy were measured using a multivariable logistic regression model, with adjusted odds ratios (aORs) and 95% confidence intervals (CIs) reported. The CSS time by race was presented as a mean with 95% CI and examined by the Kaplan–Meier method with a log‐rank test. Race associations with CSS were measured using a multivariable Cox regression model, with an adjusted hazard ratio (aHR) and 95% CI reported. Statistical analyses were conducted using the software Graph‐Pad Prism 8.0 (Graph Pad Software) and SPSS 24.0 (IBM Corp.) statistics software. A 2‐tailed *p* < 0.05 was considered statistically significant.

## 3 RESULTS

### 3.1 Population characteristics

This study's population demographic and clinical characteristics are summarized in Table 1. Of the 1,113,256 patients (mean [SD] age, 64.3 [12.1] years; 568,170 [51.0%] men), 947,900 (85.1%) were White and 165,356 (14.9%) were Black. Black patients had a significantly younger age at diagnosis than White patients. Black patients were more likely to be male and unmarried, and White patients were more likely to be insured. The cancer primary site and T, N, and M stages significantly differed by race. There were 775,606 (69.7%) patients that received surgery and 337,650 (30.3%) that did not. Black patients had a significantly lower surgical rate (62.3%) than White patients (71.0%).

**TABLE 1:** Demographic and clinical characteristics of white and black patients with 1 of 11 deadliest cancers in the US, 2007–2015.

Characteristics	All	White	Black	*P*
N (%)	1,113,256 (100.0)	947,900 (85.1)	165,356 (14.9)	
Age (years)				**<0.001**
Mean (SD)	64.3 (12.1)	64.8 (12.1)	61.4 (11.7)	
Median (range)	65 (18–117)	65 (18–117)	61 (18–108)	
Sex				**<0.001**
Female	545,086 (49.0)	469,317 (49.5)	75,769 (45.8)	
Male	568,170 (51.0)	478,583 (50.5)	89,587 (54.2)	
Marital status				**<0.001**
Married	684,096 (61.5)	610,205 (64.4)	73,891 (44.7)	
Unmarried	429,160 (38.5)	337,695 (35.6)	91,465 (55.3)	
Insurance status				**<0.001**
Insured	994,601 (89.3)	865,093 (91.3)	129,508 (78.3)	
Uninsured/ Medicaid	118,655 (10.7)	82,807 (8.7)	35,848 (21.7)	
Year of diagnosis				**<0.001**
2007	126,586 (11.4)	108,855 (11.5)	17,731 (10.7)	
2008	125,221 (11.2)	107,352 (11.3)	17,869 (10.8)	
2009	126,603 (11.4)	108,006 (11.4)	18,597 (11.2)	
2010	125,441 (11.3)	106,805 (11.3)	18,636 (11.3)	
2011	124,007 (11.1)	105,615 (11.1)	18,392 (11.1)	
2012	120,743 (10.8)	102,426 (10.8)	18,317 (11.1)	
2013	120,577 (10.8)	102,224 (10.8)	18,353 (11.1)	
2014	120,578 (10.8)	102,142 (10.8)	18,436 (11.1)	
2015	123,500 (11.1)	104,475 (11.0)	19,025 (11.5)	
Primary site				**<0.001**
Lung/bronchus	166,791 (15.0)	144,735 (15.3)	22,056 (13.3)	
Colorectal	158,641 (14.3)	135,359 (14.3)	23,282 (14.1)	
Breast	312,446 (28.1)	269,722 (28.5)	42,724 (25.8)	
Pancreas	25,141 (2.3)	21,345 (2.3)	3796 (2.3)	
Prostate	282,407 (25.4)	230,532 (24.3)	51,875 (31.4)	
Liver/IBD	13,052 (1.2)	10,457 (1.1)	2595 (1.6)	
Bladder	38,661 (3.5)	35,725 (3.8)	2936 (1.8)	
Esophagus	15,228 (1.4)	13,502 (1.4)	1726 (1.0)	
Ovary	22,899 (2.1)	20,694 (2.2)	2205 (1.3)	
Kidney/renal pelvis	60,109 (5.4)	51,653 (5.4)	8456 (5.1)	
Stomach	17,881 (1.6)	14,176 (1.5)	3705 (2.2)	
T stage				**<0.001**
T1	452,580 (40.7)	386,581 (40.8)	65,999 (39.9)	
T2	341,810 (30.7)	290,626 (30.7)	51,184 (31.0)	
T3	216,453 (19.4)	185,490 (19.6)	30,963 (18.7)	
T4	102,413 (9.2)	85,203 (9.0)	17,210 (10.4)	
N stage				**<0.001**
N0	799,451 (71.8)	682,719 (72.0)	116,732 (70.6)	
N1	173,663 (15.6)	146,897 (15.5)	26,766 (16.2)	
N2	106,786 (9.6)	90,470 (9.5)	16,316 (9.9)	
N3	33,356 (3.0)	27,814 (2.9)	5542 (3.4)	
M stage				**<0.001**
M0	961,573 (86.4)	821,096 (86.6)	140,477 (85.0)	
M1	151,683 (13.6)	126,804 (13.4)	24,879 (15.0)	
Use of surgery				**<0.001**
No	337,650 (30.3)	275,313 (29.0)	62,337 (37.7)	
Yes	775,606 (69.7)	672,587 (71.0)	103,019 (62.3)	

*Note*: Bold P‐values indicate statistically significant.

Abbreviation: IBD, Intrahepatic Bile Duct; SD, Standard deviation.

Among the 775,606 surgical patients, 246,005 (31.7%) received (neo)adjuvant chemotherapy and 215,183 (27.7%) received (neo) adjuvant radiotherapy (Table S1). The median follow‐up time was 50 months (interquartile range: 26–80), 116,290 (15.0%) died from cancer, and 659,316 (85.0%) died from other causes or were still alive.

### 3.2 Race associations with surgery use of and (neo)adjuvant therapy

**FIGURE 2:**
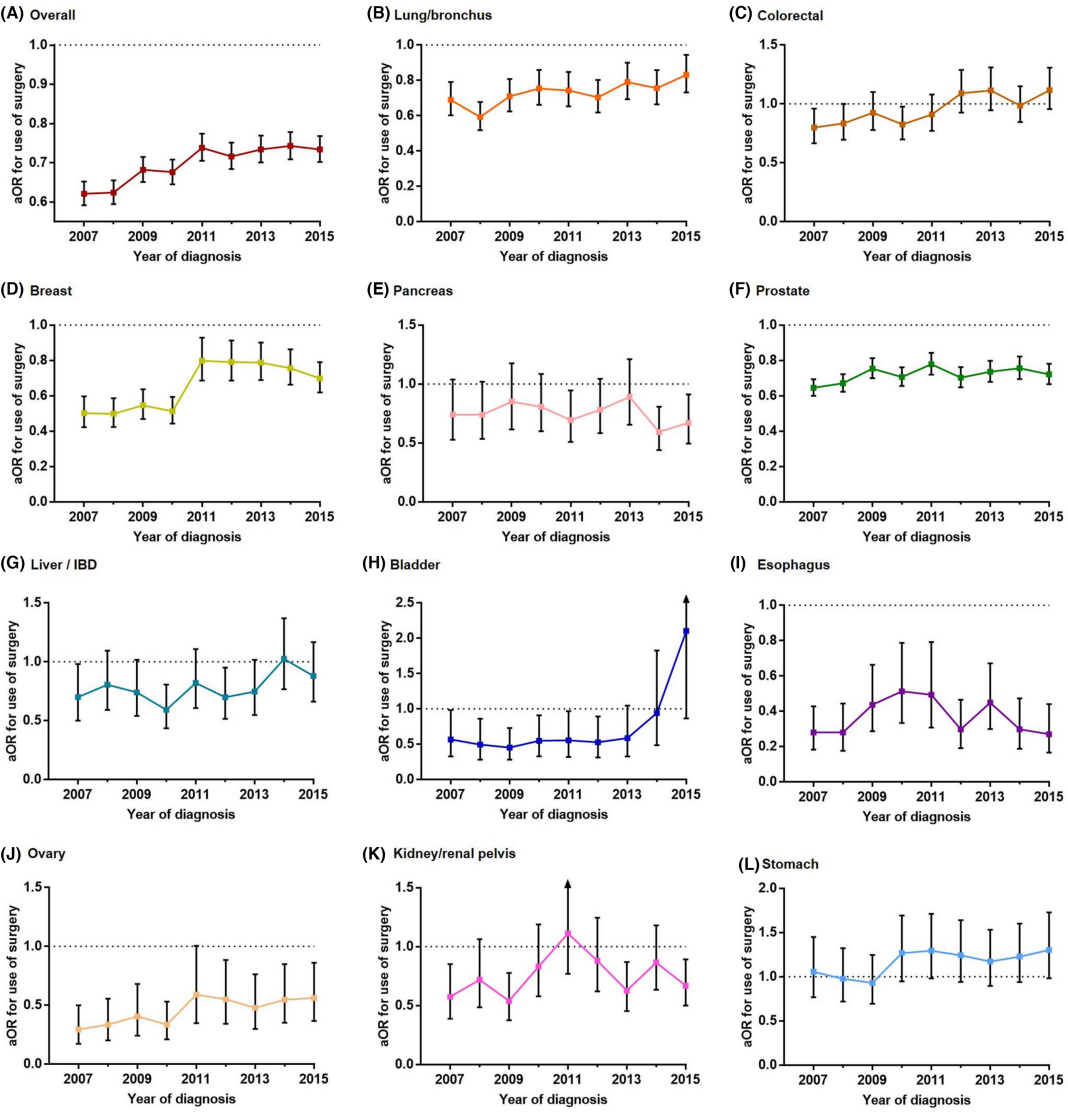
Trends in the adjusted odds ratio (aOR) of surgery use (Black vs. White) by cancer type. The aOR for overall cancer was measured after controlling for age, sex, marital status, insurance status, primary site, and T, N, and M stage. The aOR for breast, ovary and prostate cancer was measured after controlling for age, marital status, insurance status, and T, N, and M stage; the aOR for lung/bronchus, colorectal, pancreas, liver/ Intrahepatic Bile Duct (IBD), bladder, esophagus, kidney /renal pelvis, and stomach cancer was measured after additionally controlling for sex.

After entering age, gender, race, marital status, insurance status, primary site, and T, N, and M stages into a multivariable logistic regression model, it was observed that Black patients were less likely to receive surgery compared to White patients over all of the analyzed years. The Black‐White surgery use gap gradually narrowed, with the aOR increasing from 0.621 (0.592–0.652) in 2007 to 0.734 (0.702–0.768) in 2015 (Figure 2A).

For the 11 specific cancers, the Black‐White surgery use gap was continuously present in lung, breast, prostate, esophageal, and ovarian cancers. Esophageal cancer had the strongest Black‐White gap for surgery use (aOR, 0.280–0.494), followed by ovarian cancer (aOR, 0.294–0.591), breast cancer (aOR, 0.499–0.798), prostate cancer (aOR, 0.645–0.779), and lung cancer (aOR, 0.590–0.831). Except for esophageal cancer, the other 4 cancers showed a narrowed black‐white gap trend for surgery use (Figure 2).

In surgically treated patients with LN metastasis, Black patients with CRC were continuously less likely to receive (neo)adjuvant radiotherapy than white patients (Figure S1). No Black‐White gap was detected for use of (neo)adjuvant chemotherapy for any cancer type (Figure S2).

### 3.3 Race associations with CSS

**FIGURE 3:**
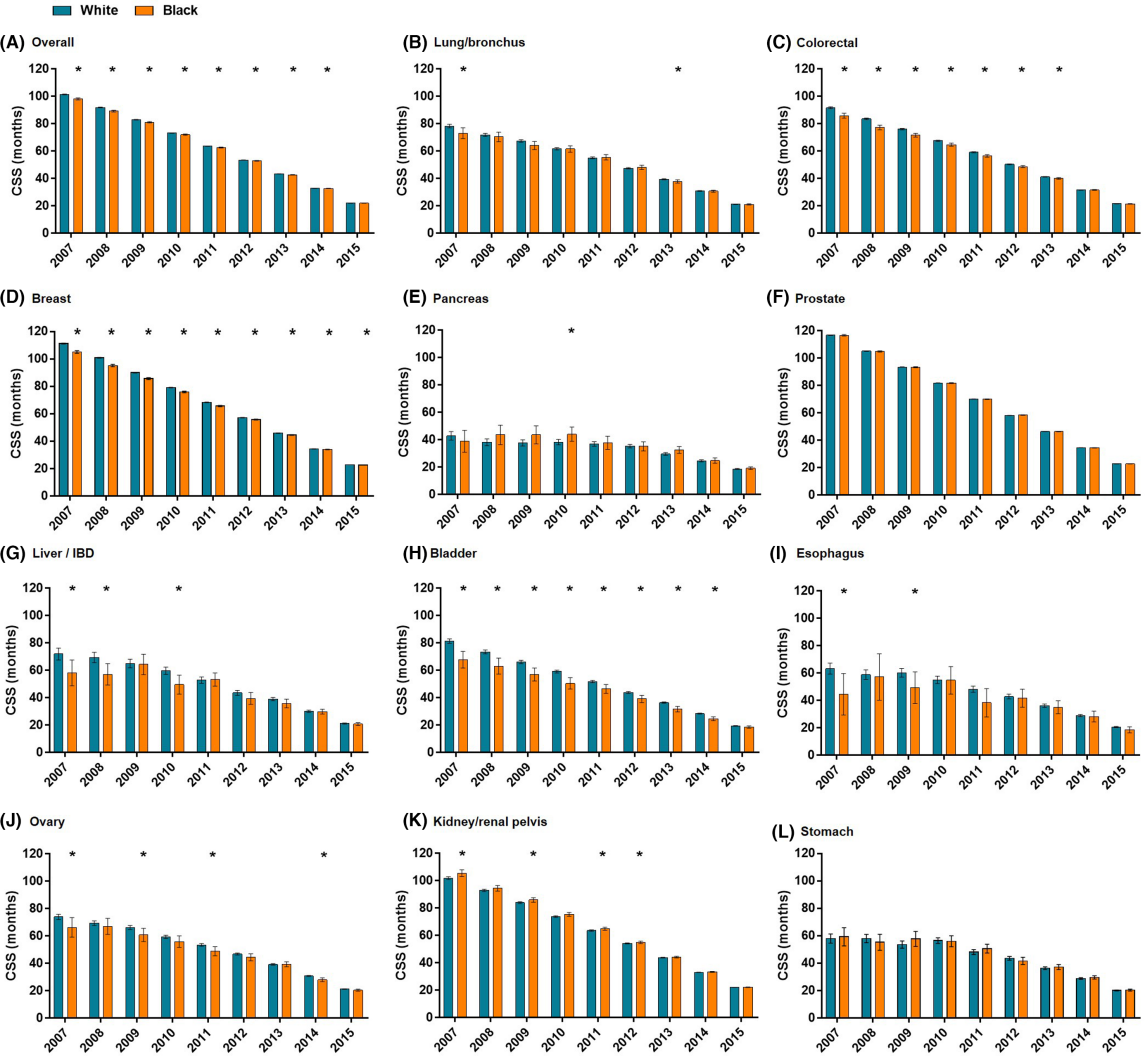
Univariable analysis of black‐white disparities in cancer‐specific survival (CSS) of patients undergoing surgery year by year. CSS time is presented by mean with 95% CI and examined by Kaplan–Meier method with a log‐rank test. *statistically significant. IBD: Intrahepatic Bile Duct.

The CSS differences between Black and White surgery patients were compared. In the univariable analysis, Black patients were more likely to have a continuously shorter CSS than White patients for colorectal, breast, and bladder cancer (Figure 3 **)**.

**FIGURE 4:**
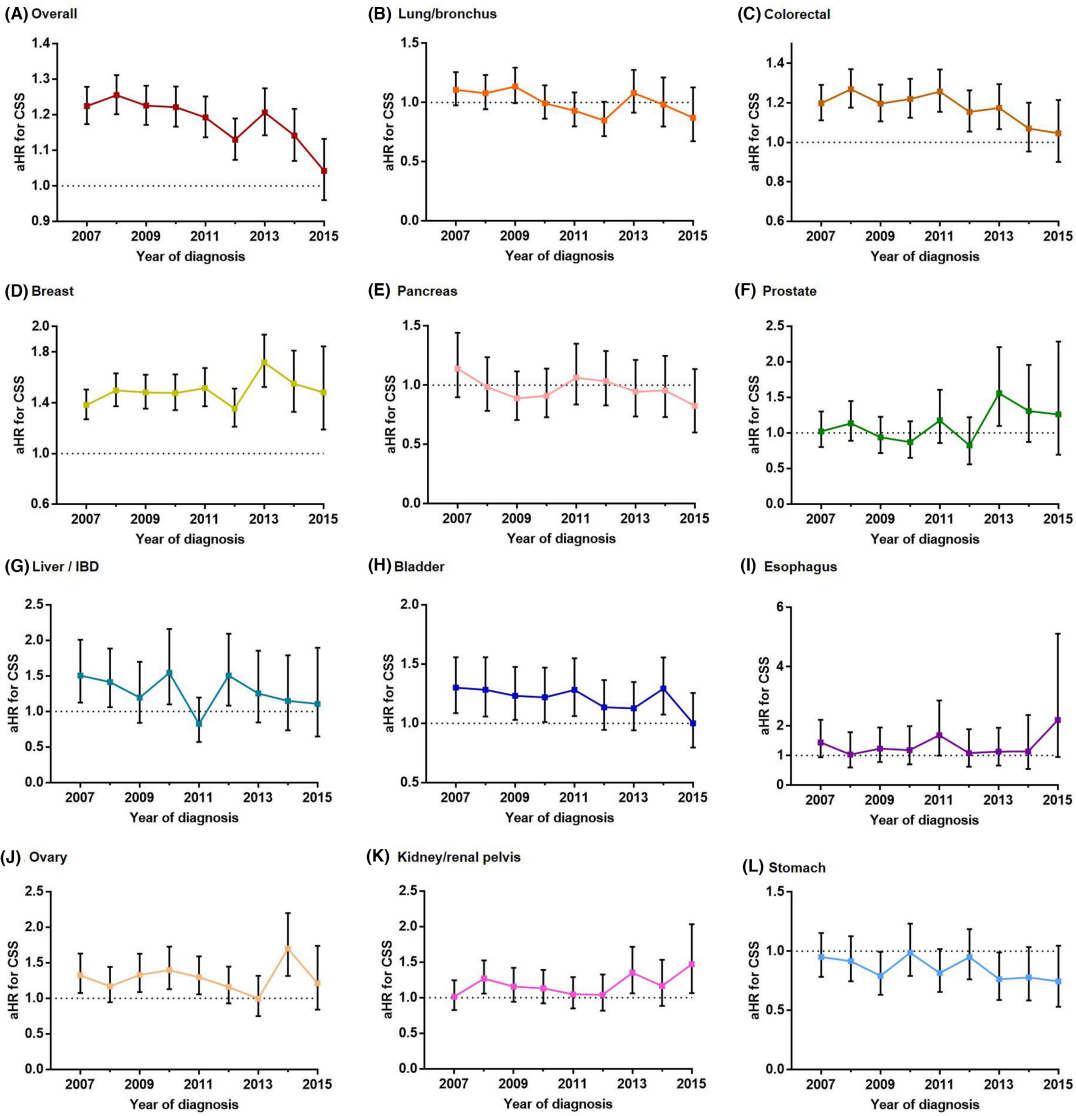
Trends in the adjusted hazard ratio (aHR) of cancer‐specific survival (CSS) (Black vs. White) in patients undergoing surgery. The aHR for overall cancer was measured after controlling for age, sex, marital status, insurance status, primary site, T, N, and M stage, (neo) adjuvant radiotherapy, and chemotherapy. The aHR for breast, ovary, and prostate cancer was measured after controlling for age, marital status, insurance status, T, N, and M stage, (neo) adjuvant radiotherapy, and chemotherapy; the aHR for lung/bronchus, colorectal, pancreas, liver/IBD, bladder, esophagus, kidney /renal pelvis, and stomach cancer was measured after additionally controlling for sex.

After adjusting for age, gender, marital status, insurance status, primary site, T, N, and M stages, and (neo) adjuvant radiotherapy and chemotherapy, Black patients who received surgery were more likely overall to have a worse CSS rate than White patients who received surgery. The Black‐White gap in CSS gradually narrowed, with the aHR decreasing from 1.224 (1.278–1.173) in 2007 to 1.042 (1.132–0.96) in 2015 (Figure 4A). A similar narrowed trend in the Black‐White CSS gap was found in patients with CRC and bladder cancer. However, the Black‐White CSS gap was continuously present and tended to extend for breast cancer.

## 4 DISCUSSION

Our population‐based, cohort study presented 3 features that distinguished it from other studies. First, most of the previous studies examining Black‐White disparities in cancer surgery have been limited to a single cancer type^6^, ^7^, ^16^, ^17^, ^18^ and lacked a full examination. Our study utilized the 11 deadliest cancers in the US to elaborate a pan‐cancer atlas and then examined the overall and specific Black‐White disparities in cancer surgery and survival. Second, our study explored Black‐White disparities regarding (neo)adjuvant radiotherapy and chemotherapy use in surgery patients, providing deeper information on cancer surgery disparities. Finally, we examined whether and how the Black‐White disparities changed from 2007 to 2015.

In the past decade, alongside ASCO's efforts to reduce racial/ethnic gaps in cancer care, a nationwide campaign against racial/ethnic disparities throughout the American healthcare system has been underway.^19^, ^20^, ^21^, ^22^, ^23^ Racial/ethnic disparities have been extensively investigated in the surgical field for non‐cancer‐related and cancer‐related procedures.^24^, ^25^, ^26^, ^27^, ^28^, ^29^, ^30^ However, recent evidence has indicated that the racial/ethnic disparities in non‐cancer surgery use among minority groups did not significantly change from 2009 to 2014, despite the efforts of Accountable Care Organizations.^31^ Black–White disparities in major non‐cancer surgical procedures even widened for several non‐cancer surgical procedures from 2012 to 2017.^32^ Our study, different from the 2 mentioned above, found that the Black–White gaps in surgical opportunity and survival narrowed over time for overall cancer. Specifically, the Black–White gap in surgery use was continuously present but narrowed in lung, breast, prostate, and ovarian cancers. Additionally, our study identified that esophageal cancer had the biggest Black‐White gap for surgery use and did not improve over time. In the future, we should especially reinforce our efforts in the 5 cancers cited above to reduce the prevailing Black–White gaps in the use of surgery.

Our study also demonstrated that, despite the use of surgery, Black patients with CRC experienced disadvantages with (neo)adjuvant radiotherapy compared to White patients. Previous studies have shown that Black patients received surgery more frequently at low‐quality hospitals,^33^ had worse specialist referral patterns,^34^, ^35^, ^36^ and often had a low socioeconomic status (SES)^37^, ^38^, ^39^ compared with White patients. These factors can hinder Black patients from receiving guideline‐concordant cancer surgery.^40^, ^41^, ^42^ In the future, attention should be directed to a guideline‐concordant (neo)adjuvant therapy for Black patients with CRC.

Additionally, our study explored Black‐White disparities regarding long‐term CSS in surgery patients. Bliton et al.^43^ considered that a lack of surgery is responsible for the Black‐White cancer survival difference. Our study demonstrated that, despite surgery, Black‐White survival gaps existed, even after adjusting for socio‐demographic factors and (neo)adjuvant therapy. These results suggested that more causes underlie Black‐White survival gaps, which deserve attention. For example, US Black patients who undergo major cancer surgery are more likely to develop postoperative complications and in‐hospital mortality than White patients.^44^ Recently, Lam et al.^45^ found that the Black‐White gap in 30‐day postoperative surgery mortality did not narrow overall in the 9 specific cancer surgical procedures from 2007 to 2016. In contrast to these results,^45^ our study demonstrated that the Black‐White gap in surgery patients' long‐term CSS narrowed over time, both overall and specifically, for patients with CRC and bladder cancer. However, the Black‐White CSS gap was continuously present for breast cancer, which requires further attention.

Overall, our results indicate that progress has been made in narrowing the Black‐White gap in cancer surgery opportunities and survival, but we must acknowledge that the Black‐White gap still persists. In addition to access to surgery, the influence of many other factors (access to screening, delay of treatment time, geographical differences, etc.) also require our continued attention. For example, multiple retrospective studies have consistently found that Black cancer patients are more likely than White patients to face delays in treatment, especially during the coronavirus disease of 2019 (COVID‐19) pandemic.^46^, ^47^ Delayed treatment time is associated with increased disease‐specific and overall mortality. Moreover, in the case of breast cancer, where there is a persistent disparity between Black and White women, mammography rates for Black women are still significantly lower than for White women.^48^, ^49^ This might be related to Black women's underinsurance or lack of coverage for medical expenses. More than 11% of non‐elderly Black people are uninsured, compared with about 8% of non‐elderly White people, according to the Kaiser Family Foundation.^50^ This result is also similar to the demographic characteristics of our study. These findings imply that the socioeconomic determinants of health have an impact on both the quality and accessibility of healthcare. A better understanding of these gaps can help to narrow them.

There were a few limitations to this study. This was a retrospective study based on information from a publicly accessible database. As a result, the results might carry selection bias. Additionally, due to the limitations of the SEER database, we were unable to obtain more detailed surgical information. For example, we know very little about the patient's preoperative evaluation and postoperative recovery. Furthermore, there is no way to fathom which medical conditions could be treated at the hospital where the patient underwent surgery.

## 5 CONCLUSION AND RELEVANCE

To our knowledge, this is the largest study to fully examine which cancers have continuously presented Black–White gaps in surgical opportunity and survival. Overall progress has been made to narrow those gaps over time. Based on our results, future initiatives to narrow Black–White disparities in surgical oncology should target (1) lung, breast, prostate, esophageal, and ovarian cancer – for which surgery use gaps were continuously detected; (2) CRC, for which a (neo)adjuvant radiotherapy use gap continuously existed; and (3) breast cancer, for which a CSS gap was continuously present. New policies and actions, based on these targets, are required to largely help achieve racial equity in surgical oncology.

### AUTHOR CONTRIBUTORS

PZ and FH contributed to the conception and design of the study. PZ and BY contributed to the literature research and graphics. PZ drafted the manuscript. The manuscript was revised by PZ, BY, FP, and FH. All authors reviewed the manuscript and approved the submitted version.

## CONFLICT OF INTEREST

All authors declare that they have no conflict of interest. All authors have read and approved the submission.

## ETHICS STATEMENT

All procedures in this study conformed to the 1964 Helsinki Declaration and its later amendments or comparable ethical standards.

## Supporting information


Table S1
Figure S1
Figure S2

## Data Availability

All data were extracted from the Surveillance, Epidemiology, and End Results database. The data used for this study are available from the corresponding authors upon appropriate request.
