# Supplementary material for: Temporal trends in Black‐White disparities in cancer surgery and cancer‐specific survival in the United States between 2007 and 2015
Source: Cancer Med. 2022 Aug 15;12(3):3509–19. doi: 10.1002/cam4.5141 (PMC9939184; doi:10.1002/cam4.5141)
Supplement: Supplementary file 1 — Table S1 Figure S1 Figure S2 [file CAM4-12-3509-s001.docx]

Supplementary table 1. Demographic and clinical characteristics of white and black patients with surgery for cancer in the US, 2007-2015.

| Characteristics | All | White | Black | *P* |
| --- | --- | --- | --- | --- |
| N (%) | 775606 (100.0) | 672587 (70.3) | 103019 (10.8) |  |
| Age (years) |  |  |  | **<0.001** |
| Mean (SD) | 63.1 (12.5) | 63.6 (12.5) | 59.8 (12.2) |  |
| Median (range) | 63 (18-117) | 64 (18-117) | 60 (18-108) |  |
| Sex |  |  |  | **0.046** |
| Female | 453559 (58.5) | 393022 (58.4) | 60537 (58.8) |  |
| Male | 322047 (41.5) | 279565 (41.6) | 42482 (41.2) |  |
| Marital status |  |  |  | **<0.001** |
| Married | 483089 (62.3) | 437137 (65.0) | 45952 (44.6) |  |
| Unmarried | 292517 (37.7) | 235450 (35.0) | 57067 (55.4) |  |
| Insurance status |  |  |  | **<0.001** |
| Insured | 700213 (90.3) | 618543 (92.0) | 81670 (79.3) |  |
| Uninsured/Medicaid | 75393 (9.7) | 54044 (8.0) | 21349 (20.7) |  |
| Year of diagnosis |  |  |  | **<0.001** |
| 2007 | 88606 (11.4) | 77644 (11.5) | 10962 (10.6) |  |
| 2008 | 87756 (11.3) | 76785 (11.4) | 10971 (10.6) |  |
| 2009 | 88660 (11.4) | 77134 (11.5) | 11526 (11.2) |  |
| 2010 | 87476 (11.3) | 75996 (11.3) | 11480 (11.1) |  |
| 2011 | 86520 (11.2) | 74858 (11.1) | 11662 (11.3) |  |
| 2012 | 84507 (10.9) | 73010 (10.9) | 11497 (11.2) |  |
| 2013 | 83446 (10.8) | 71997 (10.7) | 11449 (11.1) |  |
| 2014 | 83737 (10.8) | 72097 (10.7) | 11640 (11.3) |  |
| 2015 | 84898 (10.9) | 73066 (10.9) | 11832 (11.5) |  |
| Primary site |  |  |  | **<0.001** |
| Lung/bronchus | 52501 (6.8) | 47200 (7.0) | 5301 (5.1) |  |
| Colorectal | 147070 (19.0) | 125918 (18.7) | 21152 (20.5) |  |
| Breast | 295346 (38.1) | 256491 (38.1) | 38855 (37.7) |  |
| Pancreas | 10422 (1.3) | 9085 (1.4) | 1337 (1.3) |  |
| Prostate | 133339 (17.2) | 113125 (16.8) | 20214 (19.6) |  |
| Liver/IBD | 5402 (0.7) | 4450 (0.7) | 952 (0.9) |  |
| Bladder | 37529 (4.8) | 34745 (5.2) | 2784 (2.7) |  |
| Esophagus | 5490 (0.7) | 5187 (0.8) | 303 (0.3) |  |
| Ovary | 21369 (2.8) | 19436 (2.9) | 1933 (1.9) |  |
| Kidney/renal pelvis | 56152 (7.2) | 48310 (7.2) | 7842 (7.6) |  |
| Stomach | 10986 (1.4) | 8640 (1.3) | 2346 (2.3) |  |
| T stage |  |  |  | **<0.001** |
| T1 | 316626 (40.8) | 279616 (41.6) | 37010 (35.9) |  |
| T2 | 248276 (32.0) | 212076 (31.5) | 36200 (35.1) |  |
| T3 | 168354 (21.7) | 145255 (21.6) | 23099 (22.4) |  |
| T4 | 42350 (5.5) | 35640 (5.3) | 6710 (6.5) |  |
| N stage |  |  |  | **<0.001** |
| N0 | 579987 (74.8) | 506701 (75.3) | 73286 (71.1) |  |
| N1 | 133653 (17.2) | 113933 (16.9) | 19720 (19.1) |  |
| N2 | 50492 (6.5) | 42602 (6.3) | 7890 (7.7) |  |
| N3 | 11474 (1.5) | 9351 (1.4) | 2123 (2.1) |  |
| M stage |  |  |  | **<0.001** |
| M0 | 734808 (94.7) | 638267 (94.9) | 96541 (93.7) |  |
| M1 | 40798 (5.3) | 34320 (5.1) | 6478 (6.3) |  |
| (neo)Adjuvant chemotherapy |  |  |  | **<0.001** |
| No | 529601 (68.3) | 463133 (68.9) | 66468 (64.5) |  |
| Yes | 246005 (31.7) | 209454 (31.1) | 36551 (35.5) |  |
| (neo)Adjuvant radiotherapy |  |  |  | **<0.001** |
| No | 560423 (72.3) | 484950 (72.1) | 75473 (73.3) |  |
| Yes | 215183 (27.7) | 187637 (27.9) | 27546 (26.7) |  |
| Follow-up time, (month), Median (quartiles) | 50 (26-80) | 51 (26-81) | 47 (24-77) | **<0.001** |
| Survival status |  |  |  | **<0.001** |
| Alive or dead of other cause | 659316 (85.0) | 573467 (85.3) | 85849 (83.3) |  |
| Dead of this cancer | 116290 (15.0) | 99120 (14.7) | 17170 (16.7) |  |

Note:

IBD: Intrahepatic Bile Duct

Bold P values indicate statistically significant.


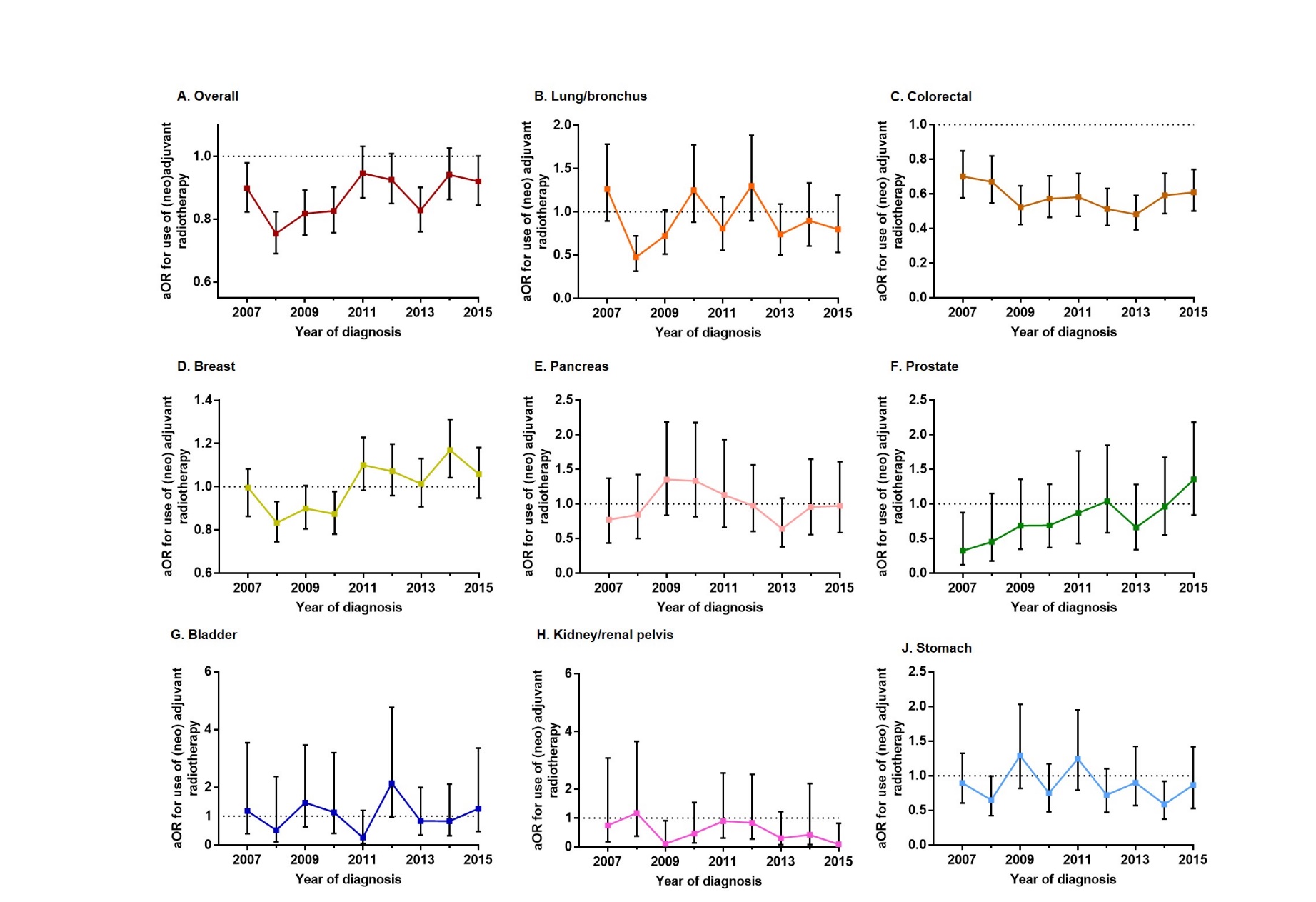


**Supplementary Figure 1. Trends in the adjusted odds ratio (aOR) of use of (neo)adjuvant radiotherapy (black vs. white) in surgically treated patients with lymph node metastasis.**

The aOR for overall cancer was measured after controlling for age, sex, marital status, insurance status, primary site, T, N and M stage.

The aOR for breast and prostate cancer was measured after controlling for age, marital status, insurance status, T, N and M stage; the aOR for lung/bronchus, colorectal, pancreas, bladder, kidney /renal pelvis and stomach cancer was measured after additionally controlling for sex.

Liver/IBD, esophagus and ovary cancer are not analyzed for limited simple size.


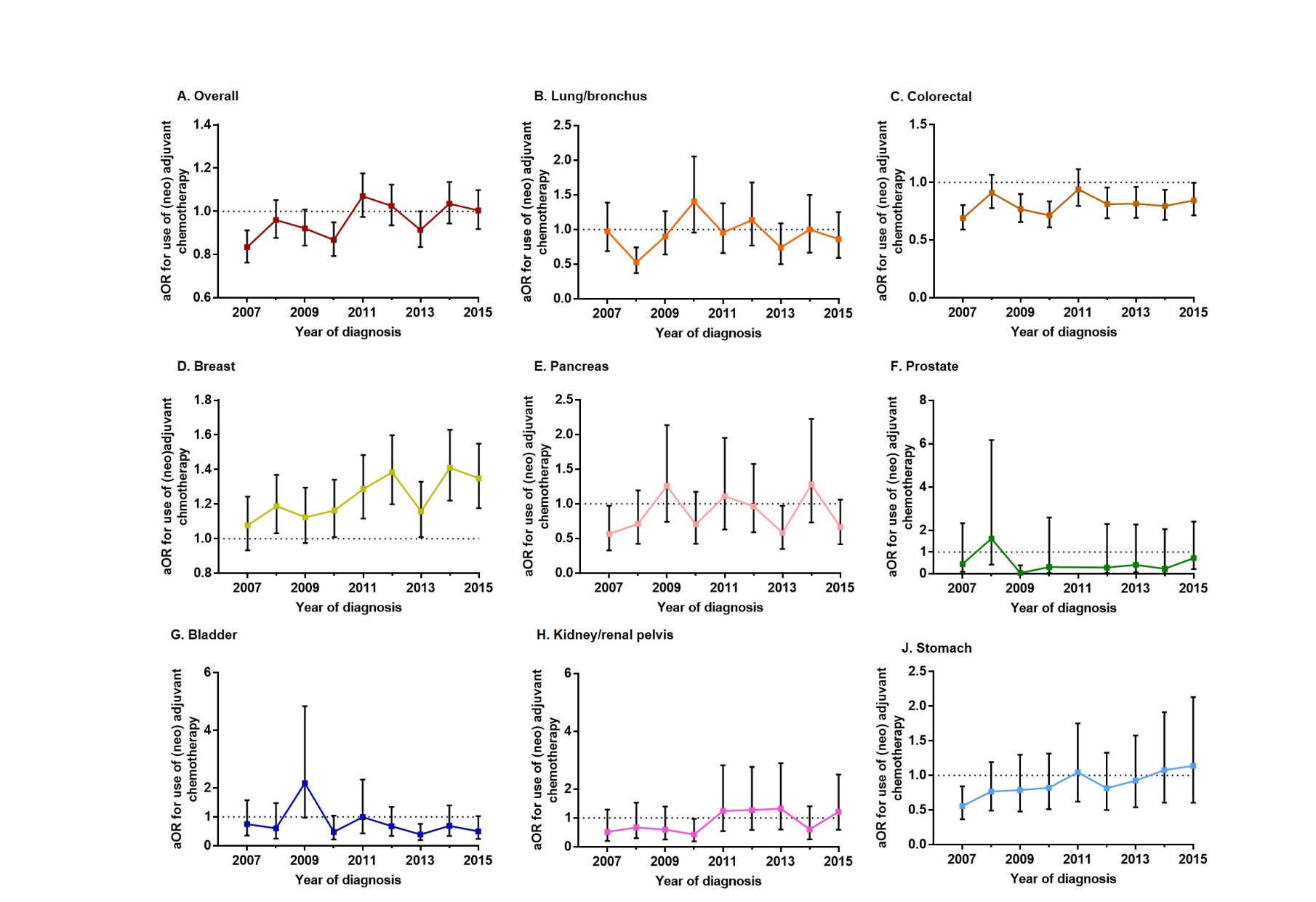


**Supplementary Figure 2. Trends in the adjusted odds ratio (aOR) of use of (neo)adjuvant chemotherapy (black vs. white) in surgically treated patients with lymph node metastasis.**

The aOR for overall cancer was measured after controlling for age, sex, marital status, insurance status, primary site, T, N and M stage.

The aOR for breast and prostate cancer was measured after controlling for age, marital status, insurance status, T, N and M stage; the aOR for lung/bronchus, colorectal, pancreas, bladder, kidney /renal pelvis and stomach cancer was measured after additionally controlling for sex.

Liver/IBD, esophagus and ovary cancer are not analyzed for limited simple size.
